# RiskDiff: a web tool for the analysis of the difference due to risk and demographic factors for incidence or mortality data

**DOI:** 10.1186/1471-2458-9-473

**Published:** 2009-12-18

**Authors:** Joan Valls, Ramon Clèries, Jordi Gálvez, Victor Moreno, Rosa Gispert, Josep M Borràs, Josepa Ribes

**Affiliations:** 1 Catalan Cancer Registry, Catalan Institute of Oncology, Barcelona, Catalonia; 2 Biomedical Research Institute, Lleida, Catalonia, Spain; 3 Department of Mathematics, Autonomous University of Barcelona, Barcelona, Catalonia, Spain; 4 Department of Clinical Sciences, University of Barcelona, Barcelona, Catalonia, Spain; 5 Bioinformatics and Biostatistics Unit, Catalan Institute of Oncology, Barcelona, Catalonia, Spain; 6 Catalan Mortality Registry, Barcelona, Catalonia, Spain

## Abstract

**Background:**

Analysing the observed differences for incidence or mortality of a particular disease between two different situations (such as time points, geographical areas, gender or other social characteristics) can be useful both for scientific or administrative purposes. From an epidemiological and public health point of view, it is of great interest to assess the effect of demographic factors in these observed differences in order to elucidate the effect of the risk of developing a disease or dying from it. The method proposed by Bashir and Estève, which splits the observed variation into three components: risk, population structure and population size is a common choice at practice.

**Results:**

A web-based application, called RiskDiff has been implemented (available at http://rht.iconcologia.net/riskdiff.htm), to perform this kind of statistical analyses, providing text and graphical summaries. Code from the implemented functions in R is also provided. An application to cancer mortality data from Catalonia is used for illustration.

**Conclusions:**

Combining epidemiological with demographical factors is crucial for analysing incidence or mortality from a disease, especially if the population pyramids show substantial differences. The tool implemented may serve to promote and divulgate the use of this method to give advice for epidemiologic interpretation and decision making in public health.

## Background

The analysis of the observed differences in the incidence or mortality of a given disease can be of great interest both for scientific and administrative purposes [1]. Studies frequently focus on comparing the number of incident or deceased cases in two given situations, with the aim of quantifying the differences observed, for further epidemiological interpretations and to give advice for decision making in public health. In this situation, time trends are usually performed to study the historical evolution of risk and to assess the occurrence of a disease in a certain period of time [2], such as comparing two different time points, in a simple trend analysis. In the same line, geographical variation of risk of a disease can be evaluated by comparing incidence or mortality rates between two areas. These comparisons are usually reported with the absolute difference in the observed number of cases (incidents or deaths) or by using the difference in the crude rates (usually per 100 000 persons), and sometimes the percentage of change is also computed [3]. Although crude rates can be used to compare different diseases in the same population, they are not useful for comparing rates of the same disease in different populations or over time [4]. To overcome this, standardized measures of risk are used to compare the evolution of risk [2], using a common reference population (the world standard population is a common choice [5,6]), and, the percentage of change of the disease is then computed [1]. However, these changes could be partially attributed to the effect of demographic factors and not only to risk, especially if the population pyramids involved in the two situations show substantial differences. A variation of the population size over time could explain variation of the number of cases, due to the consequent increment (or decrement) of persons at risk to develop or die from a certain disease. In addition, changes in the age structure between the populations involved could also lead to substantial changes in the number of cases. Regarding to this, in a number of diseases such as cancer, ageing is known to be clearly associated with molecular, cellular and physiological changes that influence carcinogenesis and subsequent cancer growth [7], and, therefore, an increase of cases among the oldest age-groups is expected [8]. In addition, another situation can arise when migration flows lead to changes in the population structure. For example, recently an increase of measles cases in Catalonia was reported, which has been partially attributed to immigration coming from undeveloped countries with poor measles vaccination coverage [9].

Bashir and Estève developed a method for partitioning the variation in the incidence or mortality from a disease between two groups, quantifying the percentage of change attributable to demographic factors (population size and structure) with respect to that which could be attributed to changes in the risk of developing or dying from a particular disease [10]. The method is based on the idea of first computing the incidence or mortality that one would have observed if the population size and structure were the same for both groups, and secondly attributing this difference with respect to the net change to demographic factors. In addition, the change attributed to demographic factors can then itself be split into that due to variation in population size and that due to changes in the population structure [10]. Thus, this method can evaluate differences in mortality (or incidence) data due to risk and demographic factors, which is not possible directly using standardized mortality (or incidence) data, since the reference population is a common standard and differences could only be attributed to risk.

The main aim of this paper is to present a set of functions in R code [11], that we have implemented, based on the method proposed by Bashir and Estève. These functions also provide convenient tables and graphical representations. In order to make these functions more widely available, we have implemented a web tool, called RiskDiff (publicly available at http://rht.iconcologia.net/riskdiff.htm) where the users can easily perform their analysis. Code for R functions is also freely available on the same web page.

Finally, to illustrate the use of this web tool, we analyse the differences in the number of deceased individuals from cancer in Catalonia in 1985 with respect to 2004, through a long period of 20 years, which is quite relevant from an epidemiological point of view.

## Implementation

The functions implemented in R are based on the method presented in the article by Bashir and Estève [10]. This method assumes two groups: the baseline (or reference) group and the comparison group. Number of incident or death cases are given for both groups, aggregated by age-groups (usually 5-years groups). The observed difference in the total number of cases or deaths between both groups can then be split into three components: cases due to changes in population size, cases due to changes in population structure (age distribution) and, for last, cases attributable to changes on the risk to develop or die due to the disease itself. Crude rates (per 100 000 people) have to be first computed, and then, the difference in the crude rates from both groups have to be partitioned in those due to risk and those due to population structure, using the following formula [10]:

Where *S*_1 _and *S*_2 _are the crude rates (per 100 000 people) for the baseline and comparison group respectively and *S*_1 _is an intermediate rate obtained for the baseline group but using the comparison group as reference population. Thus,  represents the proportional change between the observed rates in two groups, which is then partitioned in the proportional change due to population structure  and the proportional change due to differences in risk .

Two functions have been implemented in R [11]. The first one, **risk.diff()** needs four parameters called **cases.init**, **cases.end**, **pop.init** and **pop.end** which are vectors of the same length that contain the number of cases (or deaths) and the population for the two groups, for each age-group. As a result this function provides two tables that summarize the difference observed between the groups involved and a short text to facilitate interpretation. The second one, **plot.risk.diff()**, generates a graphical representation from the obtained results. These functions are available as a source text file and some examples of use are also provided. The implementation of these functions in a web interface has been made using PHP programming language [12]. Functions are executed on a remote Linux server, and results are provided on-line.

For the example illustrated in this paper, we have used cancer mortality data for the period 1985-2004 provided from the Catalan Mortality Registry. In 1985, the Catalan population was about 6 million people and near to 7 million in 2004. Population pyramids have been provided by the Catalan Statistical Institute [13]. The number of cancer deaths and the population at risk have been grouped in 5-year age bands. Registered deaths from all cancer location sites are included except those from non-skin melanoma (C44 as coded by ICD-10 [14]).

## Results

**Figure 1:**
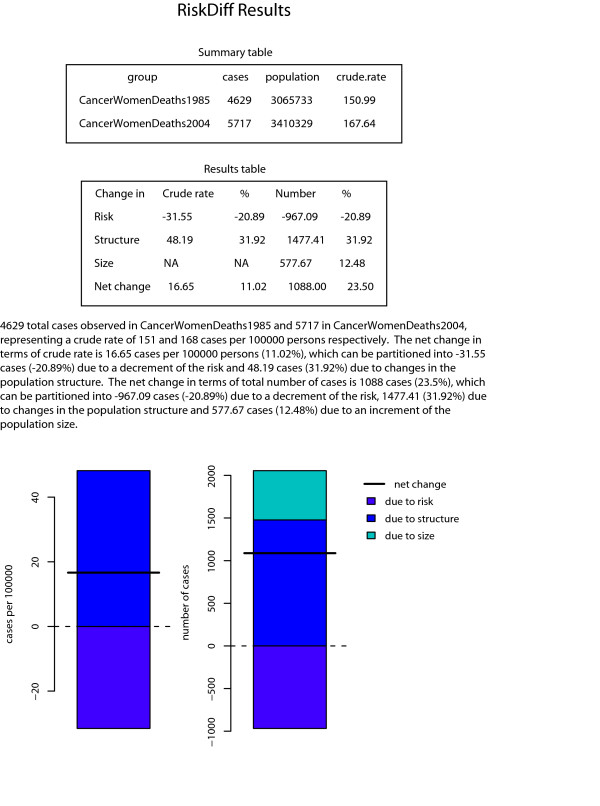
**Results obtained with RiskDiff to evaluate the change in the observed mortality for years 1985 respect to 2004, in women from Catalonia**.

**Figure 2:**
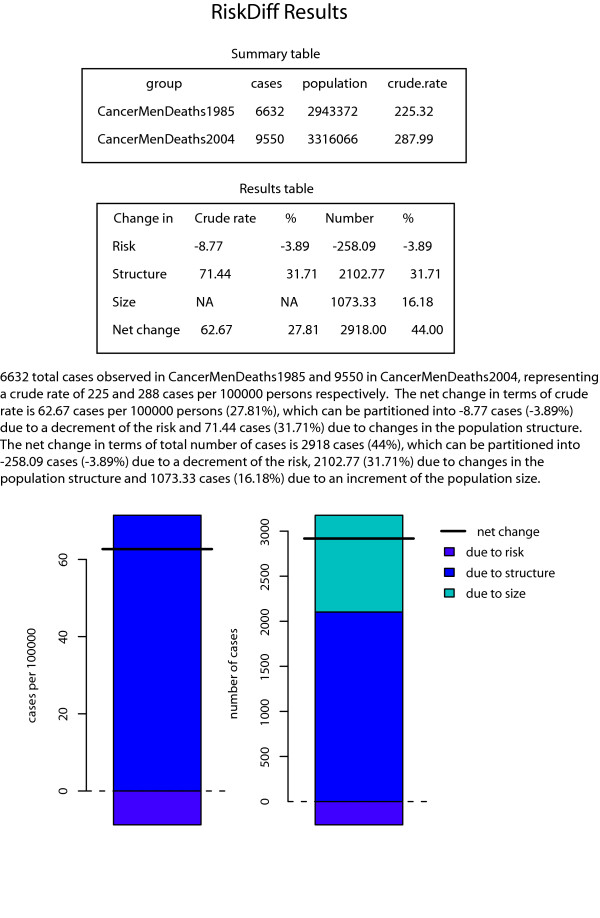
**Results obtained with RiskDiff to evaluate the change in the observed mortality for years 1985 respect to 2004, in men from Catalonia**.

The number of cancer deaths observed for both sexes in Catalonia in 1985 and 2004, and the respective Catalan population pyramids for these years are shown in tables 1 and 2. To perform the analyses with RiskDiff the user must provide four vectors with the same size containing the number of observed cases or deaths and the population in the both situations, i.e. baseline and comparison groups, for each age group. For our example mortality data from years 1985 and 2004 will be the baseline and comparison groups, respectively. Data can be plugged into RiskDiff in two ways: (1) using a tab-separated text file with 4 rows, one for each vector, with a similar structure as the one shown in table 1 and 2 or (2) directly typing the data into the web interface separately for each vector. Group labels can also be introduced in order to identify the groups. RiskDiff then produces a web page with summary tables, graphical representations and a short paragraph of text to facilitate the interpretation of the results. The results obtained when analysing mortality data from tables 1 and 2 are shown in figures 1 and 2 respectively.

**Table 1:** Number of observed deaths from cancer (all sites except non-skin melanoma) and population for years 1985 and 2004, for women in Catalonia.

	Deaths		Population (%)	
		
Age in years	1985	2004	1985	2004
0-4	6	2	163597 (5.3%)	171047 (5.0%)
5-9	7	3	230080 (7.5%)	146822 (4.3%)
10-14	6	2	247955 (8.1%)	145507 (4.3%)
15-19	10	3	238448 (7.8%)	158541 (4.6%)
20-24	16	8	228928 (7.5%)	215665 (6.3%)
25-29	13	18	220139 (7.2%)	291501 (8.5%)
30-34	49	39	207732 (6.8%)	287951 (8.4%)
35-39	96	80	207676 (6.8%)	269903 (7.9%)
40-44	94	99	181344 (5.9%)	252005 (7.4%)
45-49	147	178	167197 (5.5%)	232065 (6.8%)
50-54	261	260	189080 (6.2%)	210241 (6.2%)
55-59	363	335	180805 (5.9%)	205309 (6.0%)
60-64	451	399	164187 (5.4%)	166926 (4.9%)
65-69	567	500	133730 (4.4%)	156719 (4.6%)
70-74	685	768	117116 (3.8%)	166723 (4.9%)
75-79	722	927	92075 (3.0%)	141981 (4.2%)
80-84	667	932	59506 (1.9%)	105100 (3.1%)
85+	469	1164	36138 (1.2%)	86323 (2.5%)
Total	4629	5717	3065733 (100%)	3410329 (100%)

**Table 2:** Number of observed deaths from cancer (all sites except non-skin melanoma) and population for years 1985 and 2004, for men in Catalonia.

	Deaths		Population (%)	
		
Age in years	1985	2004	1985	2004
0-4	13	3	175499 (6.0%)	180877 (5.5%)
5-9	9	5	244280 (8.3%)	156506 (4.7%)
10-14	8	4	263451 (9.0%)	153960 (4.6%)
15-19	17	10	251663 (8.6%)	167438 (5.0%)
20-24	25	17	235813 (8.0%)	226394 (6.8%)
25-29	23	25	216962 (7.4%)	314418 (9.5%)
30-34	32	28	206881 (7.0%)	314187 (9.5%)
35-39	79	85	207612 (7.1%)	287527 (8.7%)
40-44	106	109	183591 (6.2%)	256270 (7.7%)
45-49	240	296	167220 (5.7%)	228945 (6.9%)
50-54	443	493	181749 (6.2%)	203690 (6.1%)
55-59	636	724	168626 (5.7%)	195976 (5.9%)
60-64	927	896	146463 (5.0%)	157911 (4.8%)
65-69	909	1204	103788 (3.5%)	138642 (4.2%)
70-74	1068	1527	82637 (2.8%)	134291 (4.0%)
75-79	1015	1670	58032 (2.0%)	100964 (3.0%)
80-84	706	1419	32430 (1.1%)	62208 (1.9%)
85+	376	1035	16675 (0.6%)	35862 (1.1%)
Total	6632	9550	2943372 (100%)	3316066 (100%)

Regarding the changes in the observed cancer mortality in Catalonia, a relatively high increment of both the number of deaths and crude rate is observed through the period 1985 to 2004. However, more thorough analysis reveals that the risk of dying from cancer has experienced a clear decline. More precisely, for women, the net change in the crude rate was 17 deaths per 100 000 person-years (from 151 to 168), representing an increment of 11.02%. However, our results indicate a decrease of 31.55 deaths per 100 000 person-years (21%) attributable to changes in risk while an increment of 48.19 deaths per 100 000 person-years (32%) was due to changes in population structure, i.e. ageing of the Catalan population. In terms of the absolute number of deaths, the net change was of 1088 deaths (from 4629 to 5717), representing an increment of 23.5%. In the same line, this can be partitioned into that due to an increase of the population size (577.67 deaths, 12%), that due to the ageing of the population (1477.41 deaths, 32% ) and that due to risk, which represent a decrement of 967.08 deaths (21%). Analogously, for men the net change in the crude rate was 63 deaths per 100 000 person-years (from 225 to 288), representing an increment of 27.8%. Similar to that of women, a decrement of 8.77 deaths per 100 000 person-years (4%) was attributable to changes in risk while an increment of 71.44 deaths per 100 000 person-years (32%) was due to changes in population structure. In terms of the absolute number of deaths, the net change was 2918 deaths (from 6632 to 9550), representing an increment of 44%. Once again, this can be partitioned into that due to an increase of the population size (1073.32 deaths, 16%), that due to the ageing of the population (2102.76 deaths, 32% ) and that due to risk, which represents a decrement of 258.09 deaths (4%).

**Figure 3:**
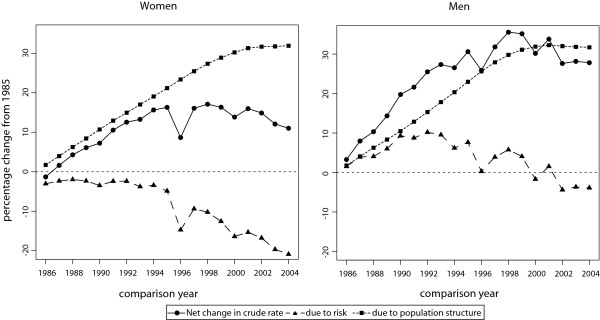
**Evolution of the differences in cancer mortality in Catalonia from 1986 to 2004 respect the baseline year 1985**.

As stated by the authors of the method, sometimes looking at two points may not be useful or may not paint a clear picture of what is actually happening [10], and they suggest that multiple comparisons can be adequate for this purpose. In these cases, one baseline group could be used, along with a set of comparison groups. This would be the case of analysing the incidence or mortality of a disease for a number of consecutive years. Even though this is not explicitly considered in RiskDiff, this analysis could actually be done by directly using the functions in R code. To illustrate this, Figure 3 shows the evolution of the percent change in terms of crude rate for the years 1986 to 2004, with respect to the baseline year 1985 (R code from this analysis is available at the web page). These results show that the real decline of the risk of dying from cancer in Catalonia started in mid 80s for women and early 90s for men. However, analysing the evolution of the net change in the crude rate, which is analogous to analyzing the evolution of the crude rate itself, indicates erroneously that mortality started to decline in posterior years and, in addition, this decline is much more clear than the one observed for the decline in the risk itself. In addition to this, these results could also give some clues for quantifying the effect of migration flows and ageing in Catalonia on the future mortality of cancer [15], which can be useful for decision making in public health.

## Discussion and conclusions

Evaluating the differences in the incidence or mortality of a disease in two given situations (such as time points, geographical areas or males versus females) without adjusting by the populations at risk involved, could lead to incorrect results [10]. Thus, it is necessary to take into account demographic factors, i.e. population size and population structure, in order to more precisely attribute which part of the observed changes is due to risk. The method presented by Bashir < Estève [10] is a good solution and a common choice at practice. This work presents a publicly available web tool that performs this analysis and provides graphical summaries and tables, with the intention of contributing to the divulgation of the method and to promote its use in epidemiology and public health sciences, which may contribute to its use at an applied level.

The results obtained from the analysis of the Catalan cancer mortality are useful to illustrate the method and its application. Thus, these results give an appropriate example that supports the importance of taking into account the changes in the population, since a simple analysis would have concluded that there was an increment in the mortality for cancer in Catalonia of 23% for women and 44% for men throughout the 20 year period analysed (1985 to 2004), however by using this method it can be stated that, actually the risk of dying from cancer has been reduced by 20% for women and 4% for men, and the major reasons for the apparent net increment was the increase of the population size (12% for women and 16% for men) and ageing of society (32% for both women and men). Thus, the use of this method is highly recommended when comparing data from heterogeneous populations, which is translated into large variability between them. The effect of immigration on the assessment of risk when comparing two time periods could be ascertained through this method, as it is the case of Catalonia [13,16,17]. Similar to other regions in Europe, the stated decline of the mortality from cancer in Catalonia in this period may be due to a number of factors such as advances in cancer treatment and diagnostic techniques as well as the decrease in the prevalence of smoking habits [18], which is somewhat similar to other regions in Europe[19].

Regarding statistical issues, the method developed by Bashir < Estève [10] does not consider specific methods for assessing whether the observed differences are significant or not, so that it is unclear how this type of hypothesis could be tested. Thus, RiskDiff has to be considered just as a tool for describing mortality or incidence data. In the case of a population-based register the differences observed can be considered as the true ones and, therefore, the differences described directly refer to the differences in the population. However, in the case of sampling a general population, these differences have to be taken with caution. In a future, a non parametric procedure, such a bootstrap one, could be implemented to RiskDiff, so that a confidence interval for the observed differences could then be provided.

In conclusion, analysing incidence or mortality data without taking into account demographic effects, can lead to results that are not easily usable for policy making. In this situation, data on the absolute number of cases and demographic determinants is highly relevant for planning purposes and for assessing future needs. This work supports the idea of combining epidemiology with demography when performing statistical analysis on the incidence or mortality from a disease, especially in dynamic populations that are affected also by other risk factors as well, that may also vary across time, gender or geographic regions.

## Availability and requirements

**Project name**: RiskDiff

**Project home page**: The webtool can be used through the following website, http://rht.iconcologia.net/riskdiff.htm. In addition, files for the R functions and examples of use can be are available as supplementary material (Additional file 1) and can also be downloaded from the web site.

**Operating system**: Platform independent for accessing the public web server

**Programming language**: R and PHP

**Requirement**: R statistical software available at http://www.r-project.org/ is required for the functions implemented.

**License**: None

**Any restriction to use by non-academics**: None

## Competing interests

The authors declare that they have no competing interests.

## Authors' contributions

JV, JR and RC initially conceived the tool and were involved in its design. JV did the statistical analysis and the implementation in R. JG implemented the web interface. All authors have been involved in drafting the manuscript and revising it critically. All authors approved the final version.

## Pre-publication history

The pre-publication history for this paper can be accessed here:

http://www.biomedcentral.com/1471-2458/9/473/prepub

## Supplementary Material

Additional file 1 **R code for the functions and examples of use**. A compressed (zip) file which contains: one file with the R code for the functions that perform riskdiff analysis (RiskDiff.R); one file with R code with examples of use of the functions (ExampleRiskDiff.R); three text files (tab-separated) that contain the data from cancer mortality in Catalonia used in the paper and that can be used as example for the R functions (Mortality.cancer.catalonia.men.1985.2004.txt, Mortality.cancer.catalonia.women.1985.2004.txt and CancerMortalityCataloniaData.txt). Click here for file
